# Application of Indoor Positioning Systems in Nursing Homes: Enhancing Resident Safety and Staff Efficiency

**DOI:** 10.3390/s24186099

**Published:** 2024-09-20

**Authors:** Chia-Rong Lee, Edward T.-H. Chu, Min-Jing Sie, Li-Tsai Lin, Mei-Zhen Hong, Ching-Chih Huang

**Affiliations:** 1 General Education Center, National Yunlin University of Science and Technology, Yunlin 640301, Taiwan; leecr@yuntech.edu.tw; 2 Computer Science and Information Engineering, National Yunlin University of Science and Technology, Yunlin 640301, Taiwan; minjing.sie@gmail.com; 3 Yunlin Veterans Home, Yunlin 64044, Taiwan; vhyli035@mail5.vac.gov.tw (L.-T.L.); vhyli064@mail5.vac.gov.tw (M.-Z.H.); vhyli023@mail5.vac.gov.tw (C.-C.H.)

**Keywords:** indoor positioning system, nursing home, long-term care facility, big data analysis, smart healthcare

## Abstract

Providing a safe and secure living environment for residents that is supported by a dedicated healthcare team is one of the core values of nursing homes. Nursing homes must protect residents from the risk of going missing, track quarantined residents and visitors to control the spread of infection, and maintain proactive nursing rounds. However, recruiting and retaining qualified caregivers and medical staff has long been a challenge. Therefore, using advanced technology to ensure the safety and security of residents is highly desirable. In this work, we first demonstrate the applicability of indoor tracking applications in a nursing home, such as resident and asset tracking, nursing assistant management, visitor tracking, infection control, and vital-sign monitoring. To monitor the locations of residents and staff, Bluetooth tags were used, providing real-time data for location tracking. We then conduct a series of quantitative analyses to illustrate how indoor tracking data can support the management of nursing homes, including characterizing residents’ activities in daily living and assessing the performance and workload of nursing assistants. Finally, we use qualitative research to evaluate the acceptability of an indoor positioning system in the nursing home. The results show that the implemented indoor positioning applications can improve the quality of healthcare and working efficiency, thereby providing a safer and more secure living environment for residents.

## 1. Introduction

With the continuous development of medical technology and improvements in living environments, population ageing has become a common demographic phenomenon in advanced countries and regions. In Taiwan, the proportion of elderly people aged 65 and over reached 17% in 2024, which is significantly higher than the threshold for an aged society as defined by the World Health Organization (WHO) [1]. It is expected that, by 2026, Taiwan will become a super-aged society, with at least 20% of the population being 65 or older [2]. Elderly people often encounter chronic diseases that can persist for a long time, such as arthritis, heart disease, dementia, and more. There is a strong demand for healthcare services, especially for disabled and demented elderly individuals who cannot care for themselves. Therefore, providing high-quality long-term services is crucial for the government of an ageing or super-aged society.

In Taiwan, the Long-Term Care Service Act was issued to establish a long-term care system and to ensure quality within the care and support services. With strong support and management from the central competent authority, various types of long-term services, such as home services, community-based services, institutional services, and family caregiver support services, have been incrementally provided across different counties and cities in Taiwan. In this work, we focus on nursing homes that provide elderly people with residential care, including medical monitoring and treatments, assistance with bathing, dressing, eating, medication administration, social activities, and more. A nursing home was invited to participate in this study on improving the quality of healthcare. The name of the nursing home is kept anonymous in this paper, and we use VAB to represent it. There were around 300 disabled elderly residents living in the VAB when the research was conducted. The VAB aims to maintain a high quality of healthcare and a safe living environment. However, like many other nursing homes, the VAB lacks enough manpower and faces challenges in monitoring elderly residents’ behaviors, controlling infections, and managing nursing assistants, each of which is described below.

### 1.1. Problems

First, taking care of disabled elderly individuals is labor-intensive, especially for those who have not yet been diagnosed with dementia but are already showing signs of cognitive decline. Since they do not have dementia, they are allowed to walk around different floors or buildings. However, sometimes they do not show up for scheduled activities, such as dining in the public area, taking a bath, or joining group exercises, and some may even leave the nursing home without permission. When these events occur, locating the missing residents quickly is crucial to reducing risk and ensuring their safety.

Second, infection control is a high-priority task for the nursing staff, not only during the COVID-19 pandemic but also during normal periods. Every resident loss is devastating for the nursing home. To prevent cross-infection, visitors are not allowed to enter residential floors, especially during the pandemic. However, some visitors do not follow the rules seriously. Similar behavior is also observed in residents who are quarantined; some of them leave their quarantined rooms without permission. Due to the shortage of manpower, these improper behaviors may not be detected in time and could lead to serious infections among elderly residents and staff members. Therefore, real-time tracking of visitors and quarantined residents is necessary to protect the health of everyone in the nursing home.

Third, outsourced nursing assistants are the frontline staff responsible for daily healthcare services at the nursing home. They must visit residents’ rooms regularly and perform scheduled tasks, such as medical monitoring and treatments, bathing, dressing, and more. Regular rounding is especially important at night to proactively address residents’ needs and prevent possible harm. Although nursing assistants are provided with adequate education and training related to their jobs, some of them do not make rounds regularly. Their improper behavior could be considered medical malpractice and could tarnish the nursing home’s reputation. To provide residents with high-quality healthcare services, properly supervising nursing rounds and balancing the workload are essential.

In summary, nursing homes often face three primary challenges: preventing residents from wandering away [3,4], enforcing infection control [5], and supervising staff [6,7]. These issues, which involve resident safety, health protection, and care quality, are prevalent in elder care settings and significantly impact both facility management and residents’ well-being. Addressing these challenges is crucial for ensuring optimal care and quality of life for nursing home residents. Recently, Huang et al. [8] and Luschi et al. [9] introduced a mobile indoor positioning system designed to guide patients and visitors through healthcare facilities. Additionally, Wichmann et al. [10] noted that experiments conducted in Germany using an ultrasound-based indoor positioning system demonstrated that such systems can significantly enhance the daily operations of hospitals and provide tangible contributions. However, existing work does not analyze the indoor trajectories of either patients or staff to further improve healthcare quality and work efficiency.

### 1.2. Contributions

To address the need for location monitoring in nursing homes, we adopted a Bluetooth-based tracking system that balances effectiveness with privacy concerns. The system uses ceiling-mounted Bluetooth readers to track wearable Bluetooth tags or smartwatches. The accuracy of positioning ranges from 5 to 10 m, depending on the density of deployment. Our key contribution is leveraging this system to enhance resident safety and staff efficiency. We developed supporting measures and policies to effectively use various indoor positioning applications. These applications allow for real-time tracking of residents, staff, and visitors, enabling quick responses to wandering residents, enforcing infection control measures, and helping in monitoring staff rounds. The major contributions of this work are described as follows.

1. We demonstrate the applicability of indoor tracking applications in a nursing home. These applications include resident tracking, quarantined resident tracking, nursing assistant tracking, visitor tracking, geofencing, vital-sign monitoring, and medical device tracking. The associated supporting measures and policies were also developed to introduce these applications to the nursing home.
2. We analyze the indoor trajectories of residents to understand their activities of daily living (ADLs), such as the times they leave or enter their rooms and the number of times they stay in specific areas. The analysis results can be used as a reference for the nursing home to detect anomalies in residents’ daily routines. When an anomaly is detected, a proactive healthcare service can be initiated. By enabling early intervention, this system can improve resident safety and health outcomes.
3. We also analyze the indoor trajectories of nursing assistants to study their workload and regular rounding behaviors. The analysis assesses whether different shifts, days, months, or rooms affect the time nursing assistants spend in residents’ rooms, the lounge area, and the amenity area. The results provide useful information for the nursing home in terms of being able to properly manage human resources and improve healthcare quality.
4. We conducted qualitative research to evaluate the acceptability of the indoor positioning system in the nursing home. A focus group, including a manager, a head nurse, a nurse, and a social worker, was invited for a face-to-face interview. Results show that the proposed indoor positioning applications can be used to improve the quality of healthcare and work efficiency.

The rest of the paper is organized as follows. Section 2 discusses the related work. Section 3 introduces the indoor positioning applications of the nursing home. Section 4 describes the model of resident’s ADL and Section 5 presents statistical analyses of nursing assistants’ performance. Section 6 describes a qualitative research. Finally, Section 7 contains a summary and the future work.

## 2. Related Work

### 2.1. Indoor Positioning Applications for Healthcare

Many studies have highlighted the important role of indoor positioning in healthcare applications. For instance, Huang et al. [8] and Luschi et al. [9] introduced a mobile indoor positioning system designed to guide patients and visitors through healthcare facilities. Their proposed applications seek to overcome the constraints of the global navigation satellite system. Additionally, J. Wichmann [10] reviewed thirty-eight studies on various IPS technologies, including Bluetooth Low Energy, Wireless-Fidelity, Hybrids, Radio-Frequency Identification, Ultra-Wideband, Infrared, and ZigBee, with the aim of evaluating the challenges of implementing IPS in hospitals. Moreover, K. Bregar [11] highlighted that Ultra-wideband (UWB) radio technology is a cost-effective option for range-based positioning, delivering very high accuracy and precision. This study also provided a comprehensive set of data analysis tools that enable reproducibility and serve as a foundation for further development of UWB positioning algorithms. Wichmann et al. [12] further noted that experiments conducted in Germany using an ultrasound-based indoor positioning system demonstrated that such systems can significantly enhance the daily operations of hospitals and make tangible contributions. In summary, indoor positioning systems can improve the daily operations and management of healthcare facilities. Therefore, it is evident that indoor positioning systems offer significant benefits and functionalities in healthcare applications. However, there is a lack of practical empirical studies, particularly in the area of big data analysis. To fully understand the role and impact of indoor positioning in healthcare institutions, more in-depth empirical research is necessary. Our study further explores these applications, emphasizing how tracking data analysis can enhance resident safety and staff efficiency in nursing homes.

### 2.2. Activity Recognition and Health Monitor for Elder Care

Many research efforts have focused on recognizing Activities of Daily Living (ADLs) for the elderly to improve their healthcare quality and secure their living environments. Generally, sensors are first deployed in a living environment to detect human activities and construct a wellness model. During runtime, sensor readings are used to detect or forecast anomalies through pattern recognition processes. Common activities that are recognized include eating, bathing, walking, and toileting, among others. When an abnormal behavior is detected, corresponding caregivers, family members, or telehealth practitioners are notified. Common behavior detection approaches include fixed-threshold classification and pattern recognition. Hemant Ghayvat et al. [13] and Yi-Chong Zeng et al. [14] deployed diverse sensors in an elderly living environment. For example, movement sensors were placed at the entrance door and kitchen to detect an elderly person’s movement. Additionally, electronic and electrical sensors were installed on devices such as rice cookers, televisions, and microwaves to monitor how and when they were used. Sensor readings were then utilized by wellness functions to determine if any abnormal behavior occurred. N. K. Suryadevara et al. [15] embedded sensors in a living environment not only to classify daily activities but also to detect emotions such as happiness, sadness, neutrality, and anger. Munkhjargal et al. [16] used passive infrared motion sensors and door sensors to monitor a single elderly woman. They developed a deep convolutional neural network to identify her activities. The proposed method is specifically designed for a solitary person, but identification becomes difficult when there is more than one person in the living environment, such as in a nursing home.

Some researchers have used wearable devices to monitor elderly behavior. Hua Li et al. [17] employed a six-axis motion sensor to detect falls. A pressure sensor was used to measure an elder’s weight, and a wearable health chip monitored pulse oximeter readings and heart rates. PIR (passive infrared) motion sensors were also deployed in the environment to recognize ADLs. Marija Stojchevska et al. [18] proposed a method for detecting ADLs using smartphone data enhanced by ambient sensors in smart homes. Employing an LSTM model, the approach handles simultaneous activities and utilizes unlabeled data for improved accuracy. Results show that smartphone data alone achieves over 50% accuracy, with ambient sensors boosting it by 7–8%. Fei Liang et al. [19] developed a multimodal approach for monitoring older adults’ daily activities using a wearable device and a companion robot, employing a dynamic Bayesian network for activity recognition. The system achieved high accuracy in both offline and real-time tests, reducing sensor activation and wearable device energy consumption.

Unlike previous approaches that focus on recognizing activities directly, we analyze the indoor trajectories of residents to understand when they leave or enter their rooms and how often they stay in specific areas. The results of this analysis can serve as a reference for nursing homes to detect anomalies in residents’ daily routines. When an anomaly is detected, proactive healthcare services can be initiated. Our work complements the aforementioned methods.

### 2.3. Caregiver Support and Management System

Caregivers and nursing assistants are the primary staff responsible for daily health care services. However, taking care of disabled elderly individuals can be stressful and exhausting. To reduce caregivers’ workloads, IoT (Internet of Things) and robotic technology have been adopted to develop support systems for elder care. M. Takahara et al. [20] developed a caregiver system that periodically checks residents’ sleeping conditions and provides suggestions if sleep problems are detected. S. Moharana et al. [21] designed robots for the purpose of dementia care-giving and proposed new roles and behaviors for these robots. For example, a dementia robot should be able to redirect patients’ conversations into positive distractions and customize itself based on user feedback. It is anticipated that supportive systems and robots with social skills can help reduce the workload of and pressure on nursing assistants. This work employs resident tracking, vital-sign monitoring, geofencing, and a panic button to support nursing home care.

Safety is often the primary reason for choosing nursing homes over in-home care for elderly individuals with chronic health problems. Therefore, regular rounding is crucial in proactively addressing residents’ needs and preventing potential issues. Matthew D. M. et al. conducted a systematic review of hourly rounding to enhance nursing responsiveness, finding that systematic and purposeful rounding improves patient satisfaction [22]. Goldsack et al. also suggested that a patient-centered proactive hourly rounding program can significantly reduce inpatient fall rates [23]. Porters are essential for hospital operations, handling patient and equipment transport. Existing systems often lack visibility in regard to their movements. Lee et al. [24] introduced an indoor location-based management system, named LOPS, at a hospital, providing real-time porter location data. A 5-month trial analyzed movement patterns, workload, and bottlenecks, leading to recommendations for improving porter efficiency.

We were inspired by previous research. The importance of periodic health monitoring led us to incorporate vital sign monitoring into our system. The use of robots in dementia care encouraged us to consider more adaptive and personalized approaches, even though we do not use robots. The emphasis on regular rounding to improve patient satisfaction motivated our focus on tracking and evaluating rounding practices. Our work differs by analyzing indoor trajectories to track residents’ activities of daily living, such as their movements between rooms and areas. This helps detect anomalies in routines, prompting early intervention and improving safety. Additionally, we focus on supporting and evaluating nursing assistants, which enhances both resident care and their work experience.

## 3. Indoor Positioning Applications in Nursing Homes

**Figure 1:**
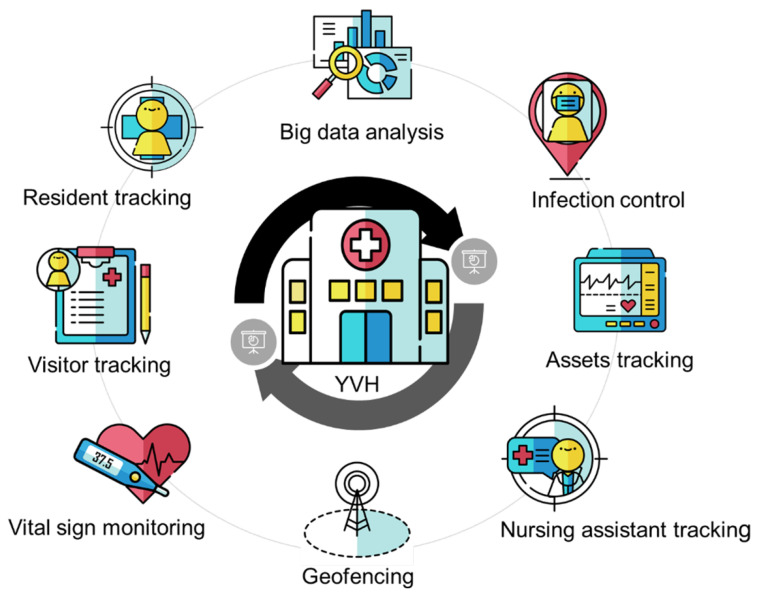
Indoor positioning applications of a nursing home.

An indoor positioning system can support various applications in a nursing home to improve healthcare quality and assist with personnel management. As shown in Figure 1, eight location-aware applications have been realized in the VAB to prove the applicability of the indoor positioning system in nursing home care. Applications include people tracking, vital-sign monitoring, geofencing, panic buttons, medical device tracking, and big data analysis. In this section, we first introduce the architecture of the indoor positioning system and its deployment. We then describe how these applications are used in the daily routines of a nursing home.

### 3.1. An Overview of the Indoor Positioning System in Healthcare

**Figure 2:**
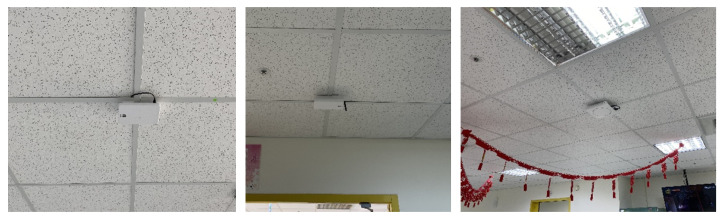
Bluetooth readers are mounted under ceilings.

**Figure 3:**
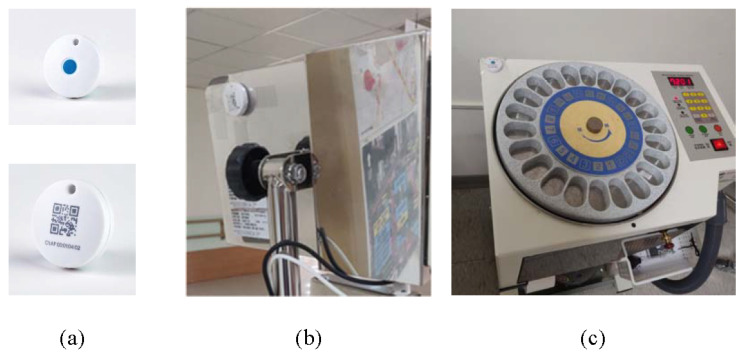
(**a**) Bluetooth tag, (**b**) Infusion pump with a Bluetooth tag, and (**c**) Automated medication dispenser with a Bluetooth tag.

**Figure 4:**
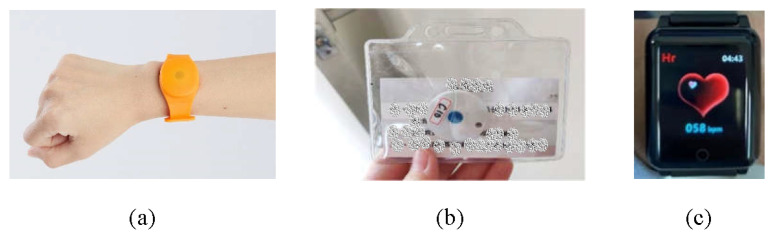
(**a**) Bluetooth wrist tag, (**b**) Bluetooth badge, and (**c**) Bluetooth smartwatch.

A Bluetooth-based indoor positioning system is used to track assets and people inside a four-floor residential care building. In our work, the required positioning accuracy depends on the location of the residents. For instance, room-level accuracy (5–10 m) is necessary when they are in their rooms, while zone-level (10–20 m) accuracy suffices in entertainment areas and near the nurse station. Floor-level accuracy (>20 m) is needed when residents are on different floors. As shown in Figure 2, Bluetooth readers were mounted under ceilings to periodically scan nearby Bluetooth tags and smartwatches. The collected device IDs and RSSI (Received Signal Strength Indicator) values are sent to a remote server, where each device’s location is determined. In addition, tracking multiple elderly residents simultaneously is necessary, which can be achieved through the unique Bluetooth IDs being assigned to each tag. Further, the system is flexible and can provide different levels of positioning accuracy, which can be set at room-level accuracy (5–10 m), zone-level accuracy (10–20 m), and floor-level accuracy (>20 m). The more densely the Bluetooth readers are deployed, the more accurate the positioning. In this work, the third floor of the residential care building is used as a demonstration floor, where the indoor positioning system provides room-level accuracy in eight selected residents’ rooms and zone-level accuracy in the entertainment areas and the nurse station. The second and fourth floors have floor-level accuracy, while the first floor has zone-level accuracy. Figure 3a shows the back of a visitor’s badge, which contains a Bluetooth tag that periodically broadcasts its device ID. As shown in Figure 3b, each device that needs to be tracked is equipped with a Bluetooth tag. Figure 4 shows the Bluetooth wristband, Bluetooth badge, and Bluetooth smartwatch used to track people. The wristband and badge are embedded with a Bluetooth tag. The panic button in the center of the Bluetooth tag can be used to trigger an alert when the user needs immediate emergency assistance. Compared to the wristband, the smartwatch can also monitor the user’s vital signs, such as body temperature, pulse rate, blood pressure, and oxygen saturation.

Since this work focuses on how an indoor positioning system can benefit a nursing home in daily routines rather than the technical details, readers interested in the technical aspects of a Bluetooth-based indoor positioning system should refer to [25].

### 3.2. Resident and Asset Tracking

Fifteen residents were selected to participate in this project. All of them required mobility aids, such as crutches or wheelchairs, to assist with walking. Most of them showed signs of cognitive decline, making it necessary to track these residents to reduce the risk of losing them and to protect their safety. Although they were not diagnosed with dementia, they might forget to wear the Bluetooth wristbands, especially after taking a bath. Therefore, we used an alternative method to track the residents, with a Bluetooth tag being placed in a badge hanging on their crutch or wheelchair. In other words, we track the movement of the aids instead of tracking the residents directly. The residents usually stayed in the lounge area or the amenity area for social activities. The indoor positioning system was also used to track the locations of quarantined residents. Whenever a quarantined resident leaves the quarantined area, an alarm light at the nurse’s station will turn on and emit a warning sound to alert the healthcare providers. For safety reasons, the light and sound will remain on until manually switched off by a user. If a resident leaves the residential care building, the system will not only alert the healthcare providers but also indicate the door through which the resident exited. This feature provides the medical staff with a clear direction to search for the missing resident, thereby reducing search times. Bluetooth tags were also used for asset tracking, particularly for devices shared among floors or departments. The tags are attached to medical devices for tracking purposes. With asset tracking support, equipment management has become easier than it was before.

### 3.3. Visitor Tracking and Infection Control

Visitors must pass several checks at the main entrance before they can enter the nursing home in order to protect residents, staff, and other visitors from COVID-19 and other serious infectious diseases. First, visitors must make an appointment prior to arrival; walk-ins are not permitted. The number of visitors is restricted to reduce traffic during serious outbreaks. Second, visitors are required to provide a record of their TOCC (Travel history, Occupation, Contact history, and Cluster history) to ensure there are no health or safety concerns. Third, visitors must pass a body temperature check and wear a mask properly. Fourth, visitors need to sign an informed consent form prior to the tracking process. Once a visitor has completed these four steps, the security guard will issue a badge associated with a unique number. The guard also records the visitor’s name, the badge number, and the start time of the visit. After receiving a badge, the visitor can enter the residential care building.

Inside the building, visitors are only allowed to stay in the designated visiting space in the lobby in order to prevent cross-infection. They are not permitted to enter the floors where residents live. Visitors are tracked by the positioning system through Bluetooth badges while they are inside the building. If a visitor enters a restricted area, such as a residential zone, the alert system will notify the staff in the lobby to take appropriate action. After visitors finish their visits, they must return their badges at the main entrance. The security guards will record their departure time and sterilize the badges. The visitors’ traces in the residential care building are stored in a server for a period of time. If there is a confirmed case associated with the nursing home in the near future, the tracking data will be used for contact tracing to identify all possible infected persons.

### 3.4. Nursing Assistant Management

Providing regular rounding is essential for responding to any emergency medical needs. To ensure that nursing assistants perform regular rounding consistently, their activities are monitored. First, every nursing assistant signed an informed consent document, which explains that a Bluetooth tag will be used to track their locations within the residential care building. The collected data will be used solely for workload evaluation, infection control assessment, contact tracing, and research purposes. Second, after a nursing assistant signs the informed consent document, they are paired with a Bluetooth tag that has a unique number. The positioning system automatically tracks their movements while they are inside the residential care building. Based on the tracking information, the positioning system generates a rounding report that lists the times a nursing assistant enters and leaves a resident’s room during a given period. This rounding report can be used for performance evaluation. Additionally, in the event of an incident, such as bedrail injuries, falls, concussions, bedsores, or missing medications, the tracking information can be used as supplementary evidence for the investigation.

## 4. Indoor Trajectory Analysis of Residents

Indoor trajectory data on residents provide useful information for exploring their activities of daily living (ADLs), such as the times they leave or enter their rooms and the number of times they stay in the entertainment area, among other factors. When an anomaly is detected, proactive healthcare services can be implemented to ensure residents’ safety and health. In this section, we first analyze the movement of residents to understand their ADLs. We then assess whether there are significant differences in the ADLs over a given period.

### 4.1. Residents’ ADLs

We conducted case studies to understand the activities of daily living of two residents: A and B. Resident A was a 70-year-old woman with diabetes, high blood pressure, and an intracranial haemorrhage. She had lived in the C3 area on the third floor for four years at the time of the research. Resident B was an 89-year-old man with prostate hypertrophy and chronic obstructive pulmonary disease. He had lived in the C3 area for three years at the time of the research. Bluetooth tags were attached to the residents’ wheelchairs to indirectly record their indoor trajectories. Residents’ trajectories from August 2020 to October 2020 were used for ADL classification. Each sample represents the daily activity of a resident and includes the times the resident wakes up in the morning (T1), starts to take a nap in the early afternoon (T2), wakes up from the nap (T3), and goes to bed (T4). Since indoor trajectory is the only information used to classify daily activities, T1 denotes the first time the resident leaves their room in the morning. Additionally, T3–T2 represent the longest period the resident stays in the room from 11 a.m. to 2:30 p.m.; periods shorter than 15 min are not considered. T4 is the last time the resident enters their room before midnight. To provide a multifaceted view, activity level indices are also included in each sample: the number of times the resident leaves their room per day (B), the number of times the resident stays in the amenity area per day (E), and the number of times the resident goes to the first floor per day (F). The amenity area is a social space where residents typically play card and board games, exercise, sing, and engage in other activities. The first floor includes a roofed corridor around the building and a courtyard. Rare visits to these areas may indicate potential health problems.

For Resident A and Resident B, the original sample size was 177. After excluding extreme samples, the effective sample size was 166. Exclusion of extreme samples refers to removing the extreme values (maximum and minimum) from the collected data and converting the remaining values into Z-scores for standardization. The purpose is to ensure the reliability and validity of the subsequent statistical results. Table 1 lists the average values. Resident A’s ADLs were very regular, with no significant differences among samples. She usually woke up around 5:37 a.m., took a nap for approximately 2 h starting at 11:50 a.m., and went to bed around 8:00 p.m. Resident B had different ADLs. As shown in Table 2, except for T4, the variances in T1, T2, and T3 were small. Statistical data indicate that Resident B was more active than Resident A. One possible reason for this difference is that Resident A suffered an intracranial haemorrhage, which can cause sudden or severe headaches and numbness in the arms or legs.

The results show that indoor trajectory data provide useful information for assessing a resident’s health status. Based on a resident’s historical data, we can set a threshold as a range of two standard deviations from the average. If an observation falls outside this range, the resident’s caregivers are notified. The range can be adjusted to a smaller value if more proactive detection is needed.

### 4.2. ADL Analysis across Different Months

In order to further understand the ADLs of Residents A and B, their daily activity indices (T1, T2, T3, and T4) for August, September, and October were analyzed using repeated measures ANOVA. As shown in Table 3, for Resident A, there is no significant difference (*p* > 0.05), indicating that she has a regular daily activity pattern. In contrast, Table 4 shows that a repeated measures ANOVA reveals statistical significance among the different months for Resident B. T1 in August is significantly later than in September and October. Additionally, T2 in September is significantly later than in August and October, and T4 in September is significantly later than in August. There is no significant difference in T3 among the different months. In summary, Resident B woke up later in August compared to September and October, took a nap later in September than in August and October, and went to bed later in September than in August. The wake-up time from the nap did not show significant differences among the months.

The results of the repeated measures ANOVA show that Resident A had a regular daily activity pattern. The average value of each ADL index can be used to detect abnormalities. For example, Resident A usually wakes up at 5:30 a.m. If the wake-up time deviates significantly from the average, such as by 30 min, a proactive nursing intervention should be triggered. On the other hand, Resident B did not follow a regular pattern in his daily activities. For such residents, a specially designed healthcare plan may be needed to ensure their safety and well-being. This case study demonstrates that trajectory analysis provides valuable insights into residents’ daily activities and offers important information to enhance the quality of healthcare.

In summary, our conclusion is based on a comprehensive analysis of both residents’ indoor trajectories and their healthcare records. While trajectory data alone do not directly reveal health conditions, they provide invaluable location information. These data can indicate whether residents are in appropriate areas at the right times and how frequently they visit different parts of the nursing home. Traditional remote healthcare methods, such as smartwatches, often struggle to proactively address location-related emergencies due to their limitations in tracking indoor movements. For example, without indoor positioning data, it can be difficult to detect if a resident is spending an excessive amount of time in the bathroom. This could indicate a fall. It can also be challenging to notice if residents are wandering, which is a potential sign of dementia. In our research, we monitor key location-related indicators, such as daily room exits and time spent in public areas. If any of these indicators differ from expected patterns, caregivers are immediately notified. By combining location and health data, we can provide personalized care and enhance resident safety.

## 5. Indoor Trajectory Analysis of Nursing Assistants

This section analyzes the indoor trajectories of nursing assistants to study their workload and regular rounding behaviors. It first describes the daily routine of nursing assistants at the nursing home. It then assesses whether different shifts, days, months, or rooms affected the time nursing assistants spent in residents’ rooms, the lounge area, and the amenity area. The analysis results provide valuable information for human resource management and healthcare quality improvement.

### 5.1. Daily Routine and Care Activities at the Nursing Home

The nursing home implemented a regular daily routine to reduce uncertainty and stress for both residents and nursing assistants. The daily activities were well structured and predictable. The C3 residents usually wake up between 5:00 and 6:00 in the morning and have their breakfast from 6:00 to 7:00 in the lounge area, where dining tables and TVs are located. During meal times, the nursing assistants help disabled or senior residents to eat, drink, or take medication. After a brief period of rest, the nursing assistants help them bathe and dress from 8:00 to 9:00. For the rest of the morning, most residents spend their time watching TV or participating in social activities in the lounge or amenity area. Meanwhile, the nursing assistants provide residents with basic care, such as taking vital signs, providing fresh water, cleaning rooms, grooming residents by brushing their hair and shaving them, and more. Every two hours, they also check residents’ diapers and turn or move bedridden residents. Lunch is scheduled at around 11:00 and lasts for one hour. The nursing assistants serve meals and assist residents who are unable to feed themselves. Residents usually take a nap in their rooms after lunch until 14:00. In the afternoon, they typically stay in the lounge or amenity area for social activities similar to those in the morning. Meanwhile, the nursing assistants again provide residents with basic care, such as taking vital signs and maintaining a clean environment. Dinner is scheduled at 17:00 and lasts for one hour in the lounge area. After a brief period of rest, the residents usually return to their rooms from 19:30 to 20:30 with the help of nursing assistants. The residents stay in their rooms overnight until 6:00 the next morning. Meanwhile, the nursing assistants conduct regular checks every two hours during the night shift.

### 5.2. Indoor Trajectory Collection and Healthcare Time

In order to collect indoor trajectory data, each nursing assistant in the C3 area was required to wear a Bluetooth tag while on duty. Each nursing assistant signed an informed consent document, which stated that the collected data would be used only for workload evaluation and research purposes. The working areas for the C3 nursing assistants include eight resident rooms (Rooms 1 to 8), one lounge area, and one amenity area. Their indoor trajectories from August 2020 to October 2020 were analyzed. A trajectory is a series of location samples, with each sample containing information on when and where a nursing assistant was during their shift; that is, x and y coordinates with a timestamp. After excluding extreme samples, we had 3125 effective samples, of which 2795 were collected in the eight rooms, while the remaining 357 were collected in the lounge and amenity areas.

Nursing assistants must perform pre-scheduled services regularly in residents’ rooms to ensure the quality of healthcare. In this work, nurse rounding (NR) time is defined as the amount of time nursing assistants spend in rooms. It is expected that, the longer the NR time, the more healthcare services the nursing assistants will provide. In addition to NR time, we also consider daily living support (DLS) time, which is the amount of time nursing assistants spend in the lounge area or the amenity area. Nursing assistants assist residents there with daily living activities, such as offering fresh water, serving meals, and feeding. During the day shift, residents spend most of their time in the lounge or amenity area. Therefore, the amount of time nursing assistants spend in the lounge or amenity area from 8:00 to 16:00 is considered DLS time. During the swing shift, residents usually stay in their rooms after 20:00. Thus, the time window to extract DLS time for the swing shift is from 16:00 to 20:00. During the night shift, residents usually stay in their rooms overnight and leave after 6:00. Therefore, the time window to extract DLS time for the night shift is from 6:00 to 8:00.

In this work, healthcare time (HT) is defined as the sum of NR time and DLS time. In the following subsections, we first analyze whether there is a statistically significant difference in HT across different shifts, months, and days. We then investigate the differences in NR time among the various rooms.

### 5.3. The Healthcare Time of Different Shifts

This subsection examines whether the healthcare time across different shifts is statistically significant. The purpose is to understand if the workload is balanced among shifts. To assess this, ANOVA was first performed to determine whether there are differences in the NR time across the three work shifts. As Table 5 shows, there is a significant difference (F = 31.031, *p* = 0.000 < 0.05). The Scheffé method was further adopted to perform post hoc comparisons. As the last column of Table 5 shows, the NR time per nursing assistant per room in the swing shift (M = 13.20) is greater than that of the night shift (M = 10.65) and the day shift (M = 9.36). After further investigation and interviews, the major reason is that the swing shift has more room service tasks than the day and night shifts, such as helping residents get into bed, cleaning bathrooms, handling trash, and so on. In contrast, the workload during the night shift is lighter because most residents are sleeping overnight.

ANOVA was also performed to determine whether there are differences in the DLS time across the three work shifts. As Table 6 shows, there is a significant difference (F = 50.442, *p* = 0.000 < 0.05). According to the Scheffé post hoc test, the DLS time per nursing assistant per room during the day shift (M = 131.73) is greater than that of the swing shift (M = 78.23) and the night shift (M = 23.63). Nursing assistants on the day shift stayed in the lounge and the amenity area longer to support residents with daily routines, such as hygiene and eating. Based on the analysis results, it is recommended to allocate additional manpower to the day and swing shifts if there is a budget to increase the headcount of nursing assistants.

### 5.4. The Healthcare Time of Different Months and Days

This subsection further examines whether the healthcare time across different months, weekdays, and weekends is statistically significant. The purpose is to understand if the nursing assistant team can deliver a consistent quality of healthcare service, which is an important indicator of nursing home quality. The analysis results could also serve as a reference when the institution evaluates the contract renewal of outsourced nursing assistants.

ANOVA was used to determine whether the daily NR time per nursing assistant in different months is statistically significant. As Table 7 shows, there is a significant difference, with F = 15.075 and *p* = 0.000 < 0.05. The Scheffé method was further applied to perform post hoc comparisons. According to the Scheffé post hoc test, the daily NR time per nursing assistant in September was 11.20 min, which is greater than that of October (10.94 min) and August (8.87 min). The daily NR time in August was the shortest because some residents were hospitalized, resulting in a lower number of residents in C3 during August compared to the other two months. ANOVA was also used to determine whether the daily DLS time per nursing assistant in different months is statistically significant. As Table 8 shows, there is no statistical significance, which indicates that the nursing assistant team delivered a similar quality of healthcare service across these months.

An independent samples *t*-test was used to determine whether the daily NR time per nursing assistant on weekdays and weekends is significantly different. As Table 9 shows, there is no significant difference (t = 0.443, *p* = 0.861). Similar results are also found in the daily DLS time, as shown in Table 10, with no significant differences (t = 0.233, *p* = 0.545). Therefore, the nursing assistant team delivered a consistent quality of healthcare service on both weekdays and weekends.

### 5.5. The Nurse Rounding Time of Different Rooms

The ANOVA was performed to determine whether the daily NR time of each nursing assistant in different rooms is statistically significant. The purpose is to understand if each room received equal amount of healthcare time. According to the results of the ANOVA, shown in Table 11, there is a significant difference (F = 67.084, *p* = 0.000 < 0.05). We further adopted the Scheffé method to perform post hoc comparisons, and the results are shown in the last column of Table 11. Let $M_{i}$ denote the average daily NR time of each nursing assistant in room *i*. As Table 11 shows, $M_{4}>M_{8}>M_{7}>M_{2}>M_{1}>M_{6}>M_{5}>M_{3}$. The results imply that residents living in Rooms 4, 8, and 7 may suffer from more severe diseases and demand more healthcare services than others. To further confirm our findings, we checked the health records of residents and found that the results aligned with our expectations. One to two residents in Room 4 had dysuria and required more healthcare services. Additionally, one resident in Room 8 had severe dementia, dysuria, and difficulty swallowing. Furthermore, a resident in Room 7, who wore a diaper, needed to be checked every one to two hours and had more medical records than others. Since the health conditions of residents among different rooms vary significantly, balancing the workload among nursing assistants is important in terms of providing better quality healthcare services. The tracking of nursing assistants provides useful and objective information for the manager in regard to optimizing human resources.

We further investigated the time each shift spent in different rooms and determined whether there was a significant difference. If the nursing assistants spend more time in a room, it is expected that the residents in that room receive more healthcare services. According to the results of the ANOVA, shown in Table 12, there is a significant difference in the total amount of time the three shifts spent in Rooms 1 to 7 (*p* < 0.05). For Room 8, the total amount of time spent by the three shifts was not significant (*p* > 0.05). The Scheffé method was further adopted to perform post hoc comparisons. The results show that residents in Rooms 1, 3, 5, and 6 received more time from day shift nursing assistants than from night shift nursing assistants. To investigate the reasons behind this, we checked the chronic diseases and conditions of the residents. Residents in Rooms 1, 3, 5, and 6 were relatively healthy and did not require additional help to get in and out of bed or walk. Additionally, they did not need assistance with toileting. Therefore, the night shift spent less time in these rooms. On the other hand, the residents of Rooms 2, 4, and 7 had more time dedicated to them by the swing and night shifts. The medical records show that residents in these rooms have relatively severe chronic diseases. Residents in Room 2 suffered from moderate dementia and needed help getting in and out of a wheelchair or moving around. Residents in Room 4 needed catheter care, requiring nursing assistants to check and change catheter bags regularly. Some residents in Room 7 suffered from severe dementia and lived with a feeding tube and a catheter bag. Therefore, more care was required when these residents were resting or sleeping in their rooms, especially during the evening and overnight hours. Since the swing and night shifts spent more time in Rooms 2, 4, and 7, they spent relatively less time in other rooms. The manager may consider moving residents with severe chronic diseases to different rooms to allow nursing assistants to monitor residents with mild and severe chronic conditions simultaneously. The analysis shows that the trace information can objectively reflect the healthcare time for each room on a shift basis. Useful insights can be provided to the manager to optimize human resources.

## 6. Qualitative Feedback of Users

An interview was conducted to collect qualitative feedback from users of the indoor positioning system. The participants included a manager, a head nurse, a nurse, and a social worker. Our findings are organized into the following categories: resident management, nursing assistant management, visitor management, and vital-sign monitoring. Additionally, the qualitative data are coded as (IR-YYMMDD-#), where IR stands for interview record, YYMMDD represents the date, and # is the serial number of the interviewee. ’L’ denotes a leader and ’N’ denotes a nurse.

### 6.1. Tracking Residents and Reducing Caregiver Workload

While residents without dementia can move freely within the facility, those experiencing early cognitive decline may face challenges with finding their way and keeping appointments. This creates a difficult balance for caregivers who must prioritize both resident safety and independence. By implementing an indoor positioning system, caregivers can track residents’ locations in real time. This technology helps locate residents quickly when needed, reducing staff burden while improving safety. It allows residents to move freely, preserving their dignity and quality of life while ensuring their safety.

*“Most of our residents who have shown signs of dementia or cognitive decline are stable. However, some of them may roam around and become confused about their locations. For these residents who could become lost, we definitely need the Bluetooth tags to track their locations.”*

(IR-210105-N1)

*“I am worried about my residents when they are out of my sight. I have no clue where the missing resident went after he or she left this floor or building. I need that information.”*

(IR-210105-L1)

The interview code format is (IR-Date-Member#). ‘IR’ stands for ‘Interview’, and the ‘Date’ follows the 20YY-MM-DD format, where ‘YY’ represents the last two digits of the year. ‘N’ represents ‘Nurse’, and ‘L’ represents ‘Leader’. For example, IR-210105-N1 represents Nurse #1’s interview on 5 January 2021.

### 6.2. Assessing Health Service Quality with a Positioning System

To safeguard residents and maintain high-quality care, nursing assistants must consistently check on residents and complete assigned tasks. An indoor positioning system can monitor their movements, allowing for evaluation of their performances and workloads. These tracking data are also crucial for investigating incidents such as bedrail injuries, falls, or missed medications. By providing clear evidence of staff involvement, it aids in identifying and resolving issues promptly.

*“I want to ensure our nursing assistants enter residents’ rooms on a regular basis, like every two hours. Also, if a room is frequently visited by our caregivers, I will wonder whether there is any anomaly in the room or if any additional healthcare service is required.”*

(IR-210105-L2)

### 6.3. Tracking Visitors and Enhancing Infection Control

Residents of nursing homes are typically over eighty and often have weakened immune systems, making infection control critical. An indoor positioning system enables managers and security to monitor visitor movements closely. Visitors entering restricted areas receive immediate alerts, and, in the event of an infection, the system’s tracking data help identify potential contacts, enhancing overall safety and control.

*“Not only during the current Covid-19 pandemic but also during future pandemics, like influenza outbreaks, visitors are not allowed to enter residential areas to avoid cross-infection. However, some do not follow our rules. We need the indoor positioning system to monitor the activities and movements of visitors inside our building.”*

(IR-210105-L2)

## 7. Conclusions

In this work, we demonstrated the applicability of indoor tracking applications in a nursing home. These applications included resident and asset tracking, nursing assistant management, and visitor tracking. The successful implementation of these novel applications within the organization was attributed not only to innovation but also to various factors such as participation and commitment. A set of standard operating procedures was developed to specify who would use the system, how it would be used, where it would be used, when it would be used, and how it would be maintained. An informed consent document was also provided to subjects detailing when, why, and how they would be tracked, as well as how the data would be used. To ensure that their rights were fully protected, the tracking process only began after the informed consent document was signed.

We also used tracking data to characterize residents’ activities of daily living, such as the times residents left or entered their rooms and how frequently they visited entertainment areas or the courtyard. We found that the trace information was aligned with the health conditions of the residents. The more severe a resident’s disease, the lower their activity level. Since the signs of cognitive decline are usually slow to progress, they are often overlooked. The indoor trajectory provides valuable assistance in assessing a resident’s health status. It can be used to trigger a warning, alerting care providers to deliver proactive healthcare and thereby reducing their workloads. To evaluate nursing assistants’ performance and workloads, trace information was also used to calculate the time nursing assistants spent in residents’ rooms. An analysis of variance was conducted, and the results showed that nursing assistants spent more time in the rooms of residents with more severe conditions, such as dysuria, difficulty swallowing, dementia, and so on. We also found that nursing assistants spent a similar amount of time in residents’ rooms on both weekdays and weekends. The trace-based rounding report can objectively reflect the workload of nursing assistants, providing valuable information for human resource management.

In the future, we plan to extend this work in three different aspects: infrastructure, data analysis, and new applications. First, to further enhance the safety of residents, the coverage of the indoor positioning system will be extended to outdoor areas, such as the pond, pathways, and fences, where accidents may occur. Second, more variables will be analyzed for their effect on the quality of healthcare service and workload in conjunction with trace information. Possible variables include gender, age, job tenure, education, and shift plans. The interaction among these variables will also be investigated. Finally, different novel indoor positioning-based applications will be developed, such as a location-aware caregiver app, food delivery robot navigation, and visually impaired-friendly navigation.

## Figures and Tables

**Table 1:** Daily routine of resident A from August to October 2020.

Activities	T1	T2	T3	T4	B	E	F
Month							
August	05:36	11:54	13:54	20:14	3	1	1
September	05:38	11:51	13:46	20:19	2	1	1
October	05:28	11:50	13:46	20:10	2	1	1
Average	05:36	11:47	13:49	20:19	2	1	1

**Table 2:** Daily routine of resident B from August to October 2020.

Activities	T1	T2	T3	T4	B	E	F
Month							
August	06:31	11:03	13:58	19:06	5	9	3
September	05:50	11:43	13:58	20:01	3	9	3
October	05:58	11:15	14:02	19:47	3	9	3
Average	06:06	11:20	13:59	19:38	4	9	3

**Table 3:** Analysis of ADL timing for Resident A across three months.

ADL Index	Month	Sample Size	Mean ^1^ (Unit: min)	Standard Deviations	F	Post Hoc
T1	Aug.	25	336.16	14.943	2.454 n.s. ^2^	- ^3^
	Sept.	25	336.92	12.711		
	Oct.	25	325.36	31.201		
T2	Aug.	25	714.60	24.459	0.108 n.s. ^2^	- ^3^
	Sept.	25	711.80	27.852		
	Oct.	25	712.00	18.919		
T3	Aug.	25	834.44	24.573	0.713 n.s. ^2^	- ^3^
	Sept.	25	828.56	21.058		
	Oct.	25	828.28	15.518		
T4	Aug.	25	1214.48	28.997	0.512 n.s. ^2^	- ^3^
	Sept.	25	1219.76	23.679		
	Oct.	25	1210.80	33.327		

^1^ The Mean is the average number of minutes that has passed since midnight. For example, 336.16 (min) is the average wake up time T1 of resident A in August; that is, 5:36 a.m. (=336.16/60) in the morning. ^2^ n.s. (no significance). ^3^ ”-” not need to run a post hoc test because of no significance.

**Table 4:** Analysis of ADL timing for Resident B across three months.

ADL Index	Month	Sample Size	Mean (Unit: min)	Standard Deviations	F	Post Hoc
T1	Aug.	25	391.68	53.091	5.516 **	Aug. > Sept.; Aug. > Oct.;
	Sept.	25	353.32	45.572		
	Oct.	25	358.56	33.891		
T2	Aug.	25	663.92	7.501	7.140 **	Aug. > Sept.; Aug. > Oct.;
	Sept.	25	702.84	7.735		
	Oct.	25	675.56	10.439		
T3	Aug.	25	838.60	37.301	0.110 n.s. ^1^	- ^2^
	Sept.	25	840.04	20.764		
	Oct.	25	842.16	18.716		
T4	Aug.	25	1146.56	109.919	3.754 *	Sept. > Aug.;
	Sept.	25	1201.28	38.826		
	Oct.	25	1187.92	48.179		

* *p* < 0.05; ** *p* < 0.01; ^1^ n.s. (no significance). ^2^ “-” not need to run a post hoc test because of no significance.

**Table 5:** Daily NR time per nursing assistant per room across different shifts.

Shift	Sample Size	Mean (Unit: min)	Standard Deviations	F	Post Hoc ^1^
Day	1680	9.36	9.347	31.031 ***	
Swing	547	13.20	11.660		Swing > Day;
Night	568	10.65	9.831		Night > Day;

*** *p* < 0.001 is a significant difference; ^1^ Post hoc test only shows significant differences.

**Table 6:** Daily DLS time per nursing assistant per room across different shifts.

Shift	Sample Size	Mean (Unit: min)	Standard Deviations	F	Post Hoc ^1^
Day	261	131.73	82.421	50.442 ***	Day > Night; Day > Swing
Swing	48	78.23	41.309		Swing > Night;
Night	568	10.65	9.831		

*** *p* < 0.001 is a significant difference; ^1^ Post hoc test only shows significant differences.

**Table 7:** Daily nurse rounding time (NR) per assistant across different months.

Month	Sample Size	Mean (Unit: min)	Standard Deviations	F	Post Hoc ^1^
August	893	8.87	9.908	15.075 ***	
September	1003	11.20	10.492		September > August
October	899	10.94	9.496		October > August

*** *p* < 0.001 is a significant difference; ^1^ Post hoc test only shows significant differences.

**Table 8:** Daily DLS time per nursing assistant across different months.

Month	Sample Size	Mean (Unit: min)	Standard Deviations	F	Post Hoc ^1^
August	110	110.64	104.989	0.936 n.s. ^1^	- ^2^
September	122	102.52	62.417		- ^2^
October	125	116.74	75.313		- ^2^

^1^ n.s. (no significance); ^2^ “-” not need to run a post hoc test because of no significance.

**Table 9:** Independent samples *t*-test results for daily NR time per nursing assistant on weekdays and weekends.

Day	Sample Size	Mean (Unit: min)	Standard Deviations	F	t	*p*
weekdays	2057	10.42	10.035	0.031	0.443	0.861
weekend days	738	10.23	10.074			

**Table 10:** Independent samples *t*-test results for daily DLS time per nursing assistant on weekdays and weekends.

Day	Sample Size	Mean (Unit: min)	Standard Deviations	F	t	*p*
weekdays	258	110.63	82.428	0.367	0.233	0.545
weekend days	99	108.36	80.954			

**Table 11:** Daily NR time per nursing assistant in different rooms.

Room	Sample Size	Mean (Unit: min)	Standard Deviations	F	Post Hoc ^1^
1	386	8.69	8.003	67.084 ***	
2	347	12.76	11.529		$M_{2}>M_{1}$; $M_{2}>M_{3}$; $M_{2}>M_{5}$; $M_{2}>M_{6}$;
3	388	6.41	5.016		
4	368	16.49	13.197		$M_{4}>M_{1}$; $M_{4}>M_{2}$; $M_{4}>M_{3}$; $M_{4}>M_{5}$; $M_{4}>M_{6}$;
5	409	6.66	6.490		
6	398	7.05	5.294		
7	217	13.97	12.718		$M_{7}>M_{1}$; $M_{7}>M_{3}$; $M_{7}>M_{5}$; $M_{7}>M_{6}$;
8	282	14.52	10.945		$M_{8}>M_{1}$; $M_{8}>M_{3}$; $M_{8}>M_{5}$; $M_{8}>M_{6}$;

*** *p* < 0.001 is a significant difference; ^1^ Post hoc test only shows significant differences.

**Table 12:** Daily NR time per nursing assistant in different rooms—micro aspect.

Room	Shift ^1^	Sample Size	Mean (Unit: min)	Standard Deviations	F	Post Hoc
Room 1	1	221	10.78	9.143	21.689 ***	1 > 2; 1 > 3
	2	79	4.62	3.790		
	3	86	7.05	5.571		
Room 2	1	199	9.77	10.675	18.520 ***	2 > 1; 3 > 1
	2	69	18.32	12.183		
	3	79	15.42	10.660		
Room 3	1	235	7.41	5.504	17.375 ***	1 > 3
	2	74	6.05	3.979		
	3	79	3.75	2.963		
Room 4	1	207	10.15	0.880	88.411 ***	2 > 1; 3 > 1; 2 > 3
	2	77	28.16	1.062		
	3	84	21.44	0.704		
Room 5	1	245	7.56	7.572	8.987 ***	1 > 3
	2	80	6.53	4.551		
	3	84	4.15	3.164		
Room 6	1	240	7.64	6.281	5.041 **	1 > 3
	2	77	6.82	3.751		
	3	81	5.52	2.098		
Room 7	1	166	8.05	6.508	276.860 ***	2 > 1; 3 > 1; 3 > 2
	2	36	31.19	8.363		
	3	15	38.07	4.652		
Room 8	1	167	15.15	12.598	1.124 n.s. ^2^	- ^3^
	2	55	14.60	7.485		
	3	60	12.68	8.257		

^1^ For the three shifts, 1 is day shift, 2 is swing shift and 3 is night shift. ** *p* < 0.01; *** *p* < 0.001; ^2^ n.s. (no significance). ^3^ “-” not need to run a post hoc test because of no significance.

## Data Availability

The data that has been used is confidential.
